# Novel connections and gaps in ethylene signaling from the ER membrane to the nucleus

**DOI:** 10.3389/fpls.2014.00733

**Published:** 2015-01-05

**Authors:** Young-Hee Cho, Sang-Dong Yoo

**Affiliations:** Division of Life Sciences, College of Life Sciences and Biotechnology, Korea University, Seoul, South Korea

**Keywords:** ethylene, signaling, MAPkinasekinaseKinase, CTR1, EIN2, EIN3

## Abstract

The signaling of the plant hormone ethylene has been studied genetically, resulting in the identification of signaling components from membrane receptors to nuclear effectors. Among constituents of the hormone signaling pathway, functional links involving a putative mitogen-activated protein kinase kinase CONSTITUTIVE TRIPLE RESPONSE1 (CTR1) and a membrane transporter-like protein ETHYLENE INSENSITIVE2 (EIN2) have been missing for a long time. We now learn that EIN2 is cleaved and its C-terminal end moves to the nucleus upon ethylene perception at the membrane receptors, and then the C-terminal end of EIN2 in the nucleus supports EIN3-dependent ethylene-response gene expression. CTR1 kinase activity negatively controls the EIN2 cleavage process through direct phosphorylation. Despite the novel connection of CTR1 with EIN2 that explains a large portion of the missing links in ethylene signaling, our understanding still remains far from its completion. This focused review will summarize recent advances in the EIN3-dependent ethylene signaling mechanisms including CTR1–EIN2 functions with respect to EIN3 regulation and ethylene responses. This will also present several emerging issues that need to be addressed for the comprehensive understanding of signaling pathways of the invaluable plant hormone ethylene.

## INTRODUCTION

Ethylene is a small volatile hydrocarbon gas and mediates diverse physiological responses in plant cells. The plant hormone is synthesized by a simple two-step biochemical pathway involving conversion of S-adenosyl-L-methionine (SAM) to 1-aminocyclopropane-1-carboxylic acid (ACC) and then to ethylene, which occurs in all higher plants (Dorling and McManus, 2012; Harpaz-Saad et al., 2012). Ethylene regulates a wide variety of physiological responses throughout the life of various plants. This covers physiological regulations from seed dormancy release and germination, seedling growth, vegetative organ growth and shaping, reproductive organ growth and sex determination, fruit ripening, organ senescence, and abscission to plant–microbe interactions (McManus, 2012). This diverse ethylene physiology results from the fine-tuning of ethylene production and signaling that are under the control of complex interactions among ethylene and other signaling pathways. Therefore, ethylene signaling functions and mechanisms need to be understood at the higher order of complexity integrating other signaling pathways.

**FIGURE 1:**
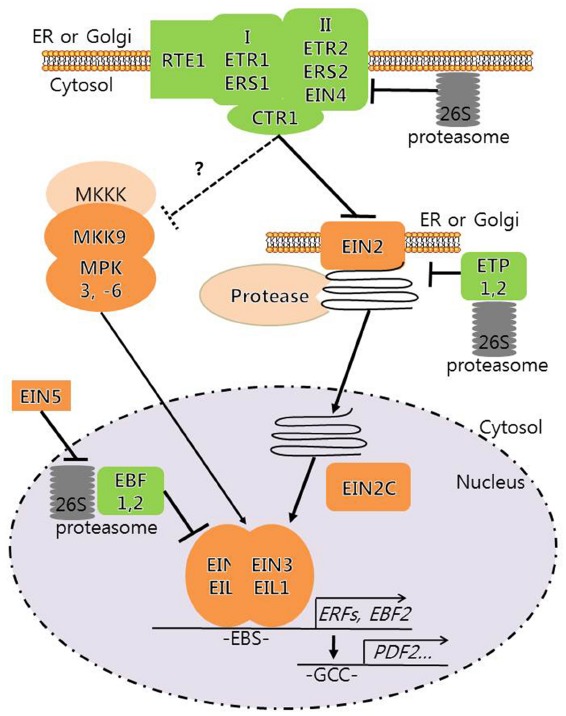
**Novel connections of ethylene signal transduction pathway in *Arabidopsis*.** The ethylene receptors comprising subfamily I (ETR1, ERS1) and II (ETR2, ERS2, EIN4) act redundantly, but also differentially, and activate a negative CTR1 protein kinase in the ER in the absence of C_2_H_4_. *Arabidopsis* RTE1 promotes ETR1 function at the membrane. EIN2 appears to act at the same ER membrane, but genetically works downstream of CTR1, implicating an assembly function of the receptor–CTR1 complex. CTR1 phosphorylates EIN2 and modulates other regulatory factors perhaps MAPKinases in the absence of C_2_H_4_. Upon ethylene signaling, the negative function of the hormone receptor–CTR1 complex is inactivated. A portion of this process includes proteasome-dependent degradation of ETR2 protein. EIN2C is cleaved from EIN2 at ER or Golgi and moves into the nucleus in the presence of C_2_H_4_. The nuclear EIN2C then activates EIN3 dependent gene transcription through a unknown mechanism. Concurrently, MKK9–MPK3,6 cascade is activated in the presence of C_2_H_4_. Both EIN2 and EIN3 protein stability is respectively under the regulation of ETP1, ETP2 and EBF1, EBF2 F-box proteins in E3 ligase complexes that are coupled with 26S proteasome activity. EIN3 accumulation in the nucleus initiates the early transcription and some of these early gene products trigger the secondary transcription. EIN5 indirectly regulates EIN3 stability through mRNA regulation of EBFs.

Ethylene is the first plant hormone, for which signaling pathway has been elucidated with mainly *Arabidopsis* genetics (Bleecker et al., 1988; Guzman and Ecker, 1990). ETHYLENE INSENSITIVE3 (EIN3) and EIN3-LIKE1 (EIL1) are the key transcription factors for ethylene immediate early gene expression (Figure 1). Protein stability regulation plays the major controlling step in the modulation of the transcription factors. EIN2, an NRAMP-like integral membrane protein located at the endoplasmic reticulum (ER), is another necessary genetic component for EIN3-dependent ethylene signaling. Recently three independent research groups (Ju et al., 2012; Qiao et al., 2012; Wen et al., 2012) have reported novel observations that EIN2 is cleaved and its processed C-terminal product (EIN2C) is translocated to the nucleus in response to ethylene. Despite the EIN2 translocation that correlates well with ethylene responses, the protease involved in EIN2 cleavage and a mechanistic function of EIN2C in EIN3-dependent gene expression have not been elucidated in these studies. Furthermore, it has yet to be examined whether or not any additional biochemical modification is required for EIN2C to be processed and translocated to the nucleus for ethylene signaling.

This focused review summarizes the new discoveries of EIN2–EIN3 process in the ethylene signaling and raises specific questions that need to be investigated for comprehensive understanding of ethylene signaling. Readers are advised to consider many excellent reviews to cover other exciting discoveries including functional modes of ethylene receptors (Hall et al., 2007; Cho and Yoo, 2009; Yoo et al., 2009; Lacey and Binder, 2014). Any newly identified EIN2–EIN3 regulatory processes will extend our understanding of integrated physiological responses of ethylene and other hormones that are involved in model and crop plant growth and development.

## ETHYLENE SIGNALING

In *Arabidopsis,* ethylene is binding to membrane proteins that are composed of five partially redundant receptors; ETHYLENE RESPONSE1 (ETR1), ETR2, ETHYLENE RESPONSE SENSOR1 (ERS1), ERS2, and EIN4. The receptors differentially express and act in different tissues and at distinct developmental stages (Bleecker et al., 1988; Chang et al., 1993; Hua et al., 1995, 1998; Hua and Meyerowitz, 1998; Sakai et al., 1998). In the absence of ethylene, ETR1 and other receptors have inverse agonistic roles in the hormone signaling and suppress ethylene responses (Hua and Meyerowitz, 1998; Wang et al., 2006). This negative action of the ethylene-free receptors is linked to another genetically identified regulator, CONSTITUTIVE TRIPLE RESPONSE1 (CTR1), which encodes a putative Raf-like mitogen-activated protein kinase kinase kinase (MAPKKK; Kieber et al., 1993; Clark et al., 1998; Huang et al., 2003). REVERSION-TO-ETHYLENE SENSITIVITY1 (RTE1) directly interacts with ETR1 in the ER membrane and Golgi-apparatus (Resnick et al., 2006; Zhou et al., 2007; Dong et al., 2008, 2010), perhaps for Cu^+^-dependent ethylene perception of the membrane receptors.

A membrane-integrated metal transporter-like protein EIN2 has been identified as a necessary component in the downstream of ethylene receptor signaling (Alonso et al., 1999). In the absence of ethylene, EIN2 is degraded by 26S proteasome activity under the action of two F-box proteins in E3 ubiquitin ligase complexes, EIN2 TARGETING F-BOX PROTEIN1 (ETP1) and ETP2 (Qiao et al., 2009). In the presence of ethylene, a part of EIN2 accumulates in plant nuclei (Ju et al., 2012; Qiao et al., 2012; Wen et al., 2012), which correlates well with the accumulation of EIN3 and EIL1 in the nucleus. Likewise, EIN3 is constantly degraded in the absence of ethylene, a process which is under the control of two F-box proteins in E3 ubiquitin ligase complexes, EIN3 BINDING F-BOX PROTEIN1 (EBF1) and EBF2 (Guo and Ecker, 2003; Potuschak et al., 2003; Yanagisawa et al., 2003; Gagne et al., 2004). The accumulation of EIN3 and EIL1 in the nucleus triggers primary transcription through EIN3-binding sites in the promoters of target genes such as *ETHYLENE RESPONSE FACTOR1 (ERF1)* and *EBF2* (Solano et al., 1998; Alonso et al., 2003; Konishi and Yanagisawa, 2008). ERF1 then itself serves as a transcriptional activator by specifically recognizing and binding to a GCC element in the promoters of ethylene secondary responsive genes (Ohme-Takagi and Shinshi, 1995; Solano et al., 1998). All the gene products of these transcription cascades lead to cellular and biochemical changes that execute the physiological and developmental adaptation of plants in response to ethylene.

Ethylene signaling feeds back to dampen ethylene responses once executed. *ERS1*, *ETR2*, and *EBF2* transcript abundance increases in response to ethylene (Hua et al., 1995; Sakai et al., 1998). These newly synthesized negative regulators in ethylene signaling may diminish and/or reset ethylene responses in the tissues that initially transduce the hormone signal.

Ethylene signaling induces gene expression and brings up the overall physiological changes of plants eventually to adapt to various biotic and abiotic stresses that trigger ethylene synthesis and signaling. For the rapid intracellular signaling, cellular protein kinases and nuclear transcription factors need to be connected instantly and dynamically. A newly identified molecular mechanism of EIN2 is the key signaling process of the ethylene immediate response and is the main point of discussion in the following section.

## EIN2 REGULATION IN ETHYLENE SIGNALING

EIN2 encodes a membrane protein with 1294 amino acids that has a hydrophobic domain at the N-terminal end (480 amino acids), containing 21% sequence identity to NRAMP metal ion transporter proteins (Alonso et al., 1999). Even so, no transporter activity has been seen with EIN2 in ethylene signaling. The EIN2 C-terminal half (840 amino acids) partially complemented the light-dependent hypocotyl response to ethylene (Smalle et al., 1997), but failed to restore the triple response that was originally used for identifying *ein2* (Alonso et al., 1999). The *ein2* mutant was also insensitive to paraquat and jasmonate (Alonso et al., 1999), but hypersensitive to ABA (Beaudoin et al., 2000; Ghassemian et al., 2000), indicating either that responses to these other signals may require EIN2-dependent ethylene signaling pathway and/or that EIN2 has multiple functions in the stress signaling responses.

The strong ethylene insensitivity of *ein2* correlates well with the diminished EIN3 protein levels in the mutant (Guo and Ecker, 2003; Wen et al., 2012). Thus, an ER membrane protein EIN2 appears to convey ethylene signaling by stabilizing the nuclear protein EIN3 through an unidentified mechanism. It has been proposed that EIN2C enters the nucleus and binds to EBF1 and EBF2 to inactivate and then stabilizes EIN3.

EIN2 has a short half-life of 30 min or less because of its degradation by the 26S proteasome activity coupled with ETP1 and ETP2 functions in E3 ligase complexes (Qiao et al., 2009). These F-box proteins interact with the C-terminal EIN2^1047–1294^ and cause degradation of EIN2 in the absence of ethylene. On the other hand, ethylene can stabilize EIN2 by diminishing ETP1 and ETP2 activity with an unknown mechanism. Null EIN2-targeting E3 ligase activity in the *etp1 etp2* double mutant causes EIN2 accumulation and leads to constitutive ethylene responses. This finding implies that EIN2 protein accumulation is necessary and sufficient for EIN3 protein accumulation and downstream ethylene responses in plant cells. However, this view has to be examined carefully with respect to the new discovery of EIN2 cleavage that is necessary for EIN2 function in ethylene signaling (Ju et al., 2012; Qiao et al., 2012; Wen et al., 2012).

Qiao et al. (2012) has reported that EIN2C, with its intrinsic nuclear localization signal (NLS), is cleaved from EIN2 and moves into the nucleus within 10 min after ethylene application. Since NLS-less EIN2 is unable to complement the loss of function *ein2* mutant phenotype in the light, EIN2C needs to be present in the nucleus to mediate ethylene signaling. EIN2 movement to the nucleus is faster than EIN2 stabilization, which mostly happens 1 to 4 h after ethylene application (Qiao et al., 2009). Protein phospho-modification analysis has revealed that Ser^645^ of EIN2 (EIN2^S645^) that is the experimentally determined amino acid residue as a cleavage site of EIN2 is the main phosphorylation and dephosphorylation site in the absence and presence of ethylene, respectively (Qiao et al., 2012). Complementation of *ein2–5* with EIN2^S645A^ (Ser to Ala) preventing phosphorylation at this residue results in induction of the EIN2C cleavage and translocation to the nucleus, and constitutive ethylene responses in the absence of ethylene. This implicates that EIN2^S645^ is phosphorylated and its phosphorylation prevents the protein cleavage and translocation to the nucleus to suppress the hormone signaling in the absence of ethylene.

Upon ethylene treatment, two different patterns of EIN2 protein accumulation were reported (Qiao et al., 2009; Ju et al., 2012). EIN2 protein levels were increased in total proteins (Qiao et al., 2009), but decreased in microsomal fractions (Ju et al., 2012). Since EIN2C movement to the nucleus plays a key role in ethylene signaling responses, observation of ethylene-dependent EIN2 protein reduction at microsomal fraction supports the signaling process. However, EIN2 accumulation in response to ethylene is a little difficult to connect to the functional mode of EIN2.

CTR1 is identified as the protein kinase responsible for phosphorylating EIN2 on several conserved residues (Ju et al., 2012). Null mutation of CTR1 displays a constitutive ethylene response and thus CTR1 acts as a negative regulator in ethylene signaling. Although CTR1 has protein domains similar to MAPKKKs, its downstream targets MAPKK and MAPK have never been identified in any plant species. Ju et al. (2012) demonstrated that CTR1 phosphorylates EIN2 at six amino acids, including S645, *in vitro*. In another report, CTR1 phosphorylates four amino acids in EIN2 (Chen et al., 2011) and the phosphorylation of two amino acid sites appears important in the ethylene signaling context. More specifically, *ein2* plants expressing either *EIN2^S645A^* or *EIN2^S924A^* conferred ethylene responses in the absence of ethylene. The lack of EIN2 phosphorylation at S645 or S645S924 was accompanied with EIN2C translocation to the nucleus and constitutive ethylene responses. It still remains to be examined whether the lack of EIN2 phosphorylation at S924 also brings about EIN2C translocation in a manner similar to the other variants. The functional complementation assays indicate that S645 and S924 phosphorylations are relevant to ethylene signaling suppression.

Recently, the discrepancy between the cleavage site of EIN2C reported by Qiao et al. (2012) and by Ju et al. (2012) was questioned (Cooper, 2013; Qiao et al., 2013). Furthermore, genetic complementation of *ein2* with *EIN2^S645A^* results in a relatively weak ethylene response compared to *EIN2^S924A^*, which implicates that EIN2S645 phosphorylation perhaps plays minor role in the hormone signaling. The clear protein accumulation pattern, precise cleavage site and major phosphorylation sites of EIN2 that are responsible for ethylene intracellular signaling are still in debates and need to be resolved.

Is EIN2C in the nucleus enough for ethylene signaling responses? Wen et al. (2012) have constructed a chimerical gene of a glucocorticoid receptor-fused to C-terminal half of EIN2 and demonstrated transgenic plants expressing the Dex-inducible *EIN2C* displayed ethylene-induced *Arabidopsis* rosette growth inhibition and also its hypocotyl growth promotion as Dex-inducible EIN2 caused EIN3 protein accumulation. Although no triple response assay was reported, *EIN2C* expression was shown to be enough to confer ethylene response in the light.

These recent studies have provided mechanistic evidence supporting the necessity of EIN2 in ethylene signaling, but how EIN2C in the nucleus modulates EIN3 function to drive the downstream physiological responses of ethylene still remains unknown. Since EIN3 fails to accumulate in *ein2* (Guo and Ecker, 2003), EIN2C most likely controls EIN3 protein stability directly or indirectly. Another important question is whether nuclear localized EIN2C is entirely sufficient for driving EIN3-dependent ethylene-response gene expression. EIN2C-complemented transgenic *ein2* lines have hitherto never been able to complement the triple response. Thus, ethylene signaling appears to require additional processes for full execution of the hormone signaling such as other CTR1-dependent and/or -independent pathways.

EIN2 interacts directly with ETR1 in the ER membrane (Bisson et al., 2009; Bisson and Groth, 2010). Fluorescence resonance energy transfer (FRET) and intrinsic tryptophan fluorescence quenching for protein interaction assays showed that ETR1 autophosphorylation is required for ETR1 and EIN2 interaction. Upon ethylene perception, ETR1 appears to be dephosphorylated to bind more efficiently to the C-terminal end of EIN2. However, only little changes of K_d_ of ETR1 and EIN2 with a very high affinity at a nanomolar scale make it difficult to substantiate its involvement in ethylene signaling. Then, the obvious question would be how to fit these interaction dynamics of ETR1 and EIN2 with ethylene-inducible EIN2C cleavage and translocation processes. Since nuclear localization of EIN2C is pivotal in ethylene signaling response, ETR1–EIN2 interaction needs to be further examined to secure its significance in ethylene signaling.

## EIN3 REGULATION IN ETHYLENE SIGNALING

The ethylene insensitivity of *ein3* is less severe than that of *etr1* and *ein2* (Chao et al., 1997). This is apparently due to its functional redundancy with EIN3–LIKE1, and thus *ein3 eil1* double mutants can block most ethylene responses as like *ein2* (Alonso et al., 2003; Binder et al., 2004).

EIN3 transcription factor protein accumulates in the nucleus in the presence of ethylene (Yanagisawa et al., 2003). In the absence of ethylene, EIN3 is negatively regulated and constantly degraded in plant cells (Guo and Ecker, 2003, 2004; Potuschak et al., 2003; Gagne et al., 2004). Such EIN3 protein degradation is controlled by 26S proteasomal activity under the regulation of two redundant F-box proteins EBF1 and EBF2. In the process, both F-box proteins bind EIN3 directly in yeast and *in vitro* systems (Solano et al., 1998; Guo and Ecker, 2003; Potuschak et al., 2003). In the *ebf1 ebf2* double mutant, EIN3 and EIL1 accumulate in the absence of ethylene and cause a seedling-arrestment phenotype (Gagne et al., 2004; Binder et al., 2007). Normal seedling growth is, however, restored in the quadruple *ein3 eil1 ebf1 ebf2* mutant indicating that EBF1 and EBF2 act more or less specifically to control EIN3 and EIL1 protein stability (Binder et al., 2007).

Apart from common functions of EBF1 and EBF2 on EIN3 and EIL1 degradation, each F-box protein has also a unique role in ethylene signaling. Individual *ebf1* and *ebf2* mutants show differential growth responses to ethylene (Binder et al., 2007) and *ctr1 ebf1* and *ctr1 ebf2* display obviously distinct phenotypes.

Although many studies have indicated that the control of EIN3 protein stability is a key regulatory process in ethylene signaling, how ethylene signaling modulates EIN3 stability in the nucleus has not been clearly elucidated. Shi et al. (2012) found that an EIN3 dependent seedling response to cold/freezing is led by EIN2 that destabilizes EBF1, causing EIN3 accumulation in the nucleus. Ethylene signaling somehow modulates protein stability of EBF1 to influence EIN3 stability. This mechanism has not been demonstrated experimentally.

Once ethylene signaling is initiated, EBF2 appears to be activated transcriptionally. Unlike *EBF1*, *EBF2* expression is induced by EIN3-dependent transcription and also by a regulatory step of mRNA stability depending on the 3^′^-untranslated region of *EBF2* in the presence of ethylene (Olmedo et al., 2006; Potuschak et al., 2006; Gregory et al., 2008). The mRNA stability of *EBF2* is under the indirect/direct control of a ribonuclease EIN5/EXORIBO-NUCLEASE4 (XNR4)/ACC INSENSITIVE1 (AIN1) activity.

EIN5 activity also controls the stability of many other RNAs including small RNAs (Olmedo et al., 2006). Therefore, it would be important to conduct further experiments to test whether or not the EIN5-dependent EBF2 regulation serves as a part of the ethylene signaling pathway.

Several studies now report that EIN3 protein levels are changed under different conditions (Lee et al., 2006; Laluk et al., 2011; Shi et al., 2012; Kim et al., 2013). In the absence of ethylene, EIN3 and EIL1 are stabilized in the light, but these proteins are degraded in the dark (Lee et al., 2006). Cold/freezing also causes EIN3 to accumulate but suppresses ethylene production, and thus the EIN3 accumulation by cold/freezing seems to be independent of ethylene signaling response (Shi et al., 2012). Furthermore, EIN3 stability regulation and plant immunity responses now point out that triple response which is one of the typical seedling responses to ethylene in the dark can be uncoupled from EIN3 accumulation in the nucleus that is a typical biochemical process in ethylene signaling. EIN3 accumulates in a null mutant *botrytis induced kinase1* (*bik1*), but this protein accumulation does not result in a triple response (Laluk et al., 2011). Instead, *bik1* is rather insensitive to ethylene. In another case, the protein level of EIN3 is down-regulated in the ectopic expression of *GDSL lipase1* (*GLIP1*; Kim et al., 2013). However, the seedlings display hypersensitivity to ethylene instead of hyposensitivity. In both cases BIK1 and GLIP1 act downstream of EIN3 but upstream of the triple response so that these mutants are interfered in EIN3 dependent responses (Liu et al., 2013; Kim et al., 2014). All these studies simply demonstrate the complexity of ethylene signaling with respect to a seemingly simple phenotypic response.

## EIN3-DEPENDENT ETHYLENE RESPONSIVE GENE EXPRESSION

Nuclear EIN3 activates primary ethylene-dependent transcription through binding to the *cis*-element AYGWAYCT within promoters of ethylene early response genes (Yamasaki et al., 2005). The DNA binding activity of EIN3, and its close homolog EIL1, often results in transcriptional activation of target genes such as *ERF1*, *AtERFs*, *ERS1*, and *EBF2* as a primary response of ethylene signaling. EIN3 also binds to the promoter regions of *PHYTOCHROME INTERACTION FACTOR1* (*PIF1*) and *PIF3* directly to activate their transcription and causes seedling greening and hypocotyl growth under lights, respectively (Zhong et al., 2012a,b).

In contrast to the well characterized transcription activator role of EIN3/EIL1, recent studies on EIN3 function have proposed transcriptional repression functions through direct binding to target gene promoters as well. EIN3 binds to the 5^′^-flanking region of *SA INDUCTION DEFICIENT2* (*SID2*) involved in salicylic acid (SA) biosynthesis (Chen et al., 2009) to negatively modulate target gene expression. Consequently, EIN3 activation compromises SA-dependent defense and causes systemic vulnerability to bacterial pathogens. Similarly, EIN3 is reported to bind to the promoter of CRT/DRE BINDING FACTOR3 (CBF3), which is a key transcription factor in cold/freezing resistance response, and suppresses its transcription (Shi et al., 2012). Thus, ethylene sensitivity seems to compromise CBF3 gene expression and cold/freezing tolerance. Such direct repressor function of EIN3 on gene expression is still rare when compared to its activator function, and needs to be further characterized more thoroughly.

Zhu et al. (2011) have investigated the interaction of jasmonate and ethylene signaling in plant defense and revealed a link between these two hormones and further investigated a molecular basis of repressor function of EIN3 in gene expression. JASMONATE ZIM DOMAIN (JAZ) transcription factors interact directly with EIN3 and suppress EIN3 transcription activity. The JAZ proteins do so in part by recruiting a histone deacetylase (HDAC6) repressor component. HDAC6 obstructs EIN3 from binding to its targeted promoters by removing acetyl groups from the histones at target chromatins. In summary, EIN3 recruits a transcriptional repressor complex to a target gene and suppresses gene expression. In the presence of both jasmonate and ethylene, JAZ destabilizes and EIN3 accumulates in the nucleus, and eventually activates target genes cooperatively. Even so, this mechanism is not enough to explain how EIN3 can act as an activator for some gene transcriptions, but as a repressor for others.

## GAPS IN OUR KNOWLEDGE OF ETHYLENE INTRACELLULAR SIGNALING

Ethylene is a key signaling molecule mediating physiological events underlying plant growth and development. Multifaceted functions of ethylene ensure developmental plasticity of plants in response to diverse environmental stress conditions. Our understanding of ethylene signaling is advanced by recent functional characterization of CTR1 phosphorylation of EIN2 and its phosphorylation status-dependent movement into the nucleus that controls EIN3 stability mediating ethylene signaling (Figure 1). Discovery of CTR1 and EIN2 connection does provide an epic moment in the field of ethylene signaling research. However, several issues and questions still remain unresolved for comprehensive understanding of ethylene signaling, including (1) which phosphorylation sites and what sizes of EIN2C are truly involved in ethylene signaling since discrepancy has been documented in the literature, (2) which protease functions in EIN2 cleavage, (3) how EIN2C manipulates EIN3 protein stability, (4) whether CTR1 is the only protein kinase that is involved in EIN2 phosphorylation, and (5) whether CTR1-dependent phosphorylation of EIN2 is sufficient for ethylene signaling. For example, a search for MAPKKs and MPKs downstream of the MAPKKK CTR1 could identify additional CTR1 substrates. MAPK cascades of MKK9–MPK3 and MPK6 also involve in EIN3 phosphorylation and its stability regulation (Yoo et al., 2008). As EIN2C cannot fully complement EIN2 functions, such a parallel pathway may exist in ethylene signaling. Taken together, our understanding of the pathways and processes of ethylene signaling is far from its completion at this stage.

Since ethylene mediates so many physiological traits important for plant-based biomass productivity and its genetic constituents in signaling pathways are largely conserved in diverse plant genomes, detailed understanding of ethylene signaling in the model plant *Arabidopsis* will provide invaluable information to screen and characterize regulatory chemicals to specifically manipulate ethylene signaling in crops and certainly be able to manipulate ethylene-dependent physiology for practical purposes.

### Conflict of Interest Statement

The authors declare that the research was conducted in the absence of any commercial or financial relationships that could be construed as a potential conflict of interest.
